# Open Barrier Membrane Technique for the Treatment of Oroantral Communications: Two Case Reports

**DOI:** 10.7759/cureus.63854

**Published:** 2024-07-04

**Authors:** Ralitsa V Yotsova, Georgi Y Papanchev, Madlen Ali, Tsvetalina Gerova-Vatsova

**Affiliations:** 1 Department of Oral Surgery, Medical University of Varna, Varna, BGR; 2 Department of Periodontology and Dental Implantology, Medical University of Varna, Varna, BGR

**Keywords:** oroantral fistula, ridge preservation, open barrier technique, dense polytetrafluoroethylene membrane, oroantral communication

## Abstract

Oroantral communications (OACs) are relatively common complications after extractions of maxillary posterior teeth. Some defects can heal spontaneously, while others require surgical treatment. The lack of an appropriate therapeutic approach can lead to the epithelialization of the OAC that causes a permanent connection between the two cavities, called an oroantral fistula (OAF), and subsequent chronic sinusitis. Various treatment modalities have been used in cases of OACs, including advancement flaps, bone grafts, synthetic materials, and barrier membranes. We present two cases of closure of OACs with dense polytetrafluoroethylene (d-PTFE) membranes (of FDI tooth #28 in the first case and #17 in the second case), which were left exposed to the oral cavity. In both cases, healing was uneventful.

## Introduction

An oroantral communication (OAC) is an unnatural opening between the oral cavity and maxillary sinus. It is a relatively common finding after extracting maxillary posterior teeth, with a reported incidence of 11% [1]. Prerequisites for OAC are some anatomical features, such as proximity of the root apices within the sinus, periapical pathology involving the floor of the sinus, traumatic extraction, and other iatrogenic causes. The lack of an appropriate therapeutic approach can lead to the epithelialization of the OAC that causes a permanent connection between the two cavities, called an oroantral fistula (OAF), and subsequent chronic sinusitis. The formation of OAF usually occurs at least 48-72 hours after the extraction when the perforation remains untreated [1].

A defect with a diameter below 3 mm can heal spontaneously if an infection does not occur [2,3]. In the case of а communication larger than 3 mm, shallow sockets, and missing socket bony walls, the blood clot can be easily dislodged, and the chances of spontaneous wound healing decrease [4]. Such cases require surgical treatment of the OAC. Various techniques have been described in the literature, the most common of which are the methods that utilize surgically advanced flaps. Other techniques require bone-substitute materials, synthetic materials, and barrier membranes [1].

Dense polytetrafluoroethylene membranes (d-PTFE) are non-resorbable, biologically inert, and biocompatible. They preserve their structural integrity during the whole healing period. Their major advantages are safety, impermeability, and easy handling. These membranes have been recently utilized for socket sealing and alveolar ridge preservation.

We present two case reports in which we used d-PTFE membranes for the management of OACs. The aim of this study is to demonstrate a relatively new, efficient, and promising method for the closure of OACs without flap mobilization.

## Case presentation

Case 1

**Figure 1:**
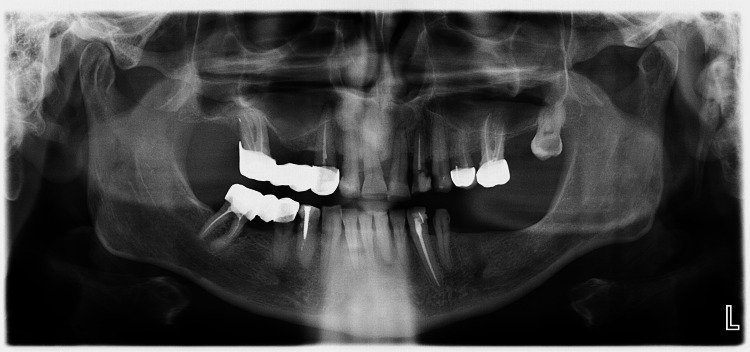
Preoperative panoramic radiograph (Case 1)

A 45-year-old patient was referred to the Department of Oral Surgery in the University Medical-Dental Centre of the Medical University of Varna, Varna, Bulgaria, in February 2024 for dental rehabilitation before upcoming chemotherapy. The patient had been diagnosed with multiple myeloma IgG λ, stage I ISS. Panoramic radiography was carried out, and extractions of the compromised teeth (FDI teeth #13, 16, 21, 24, 28, 31, 41, and 45) were scheduled (Figure 1).

**Figure 2:**
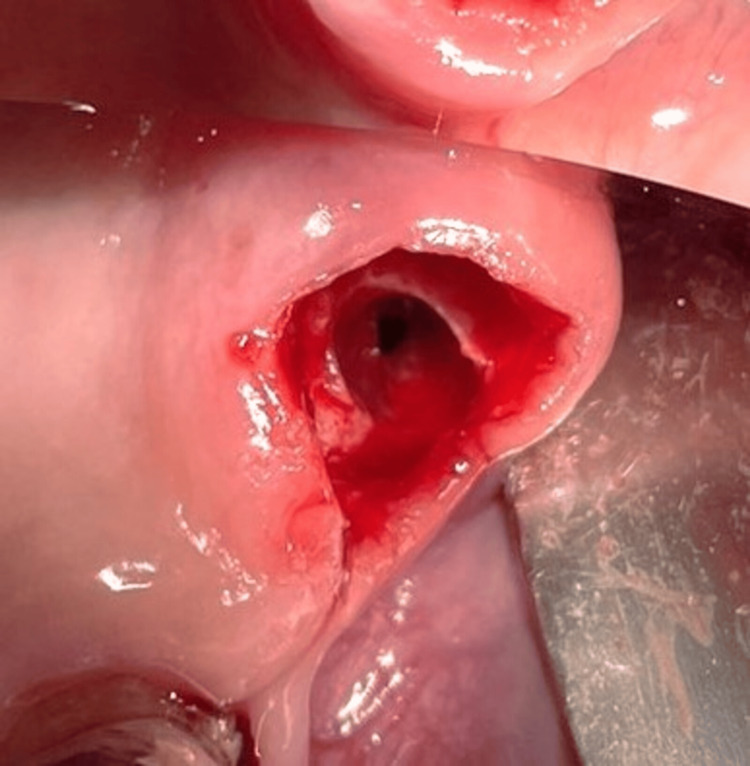
Oroantral communication: clinical view (Case 1)

**Figure 3:**
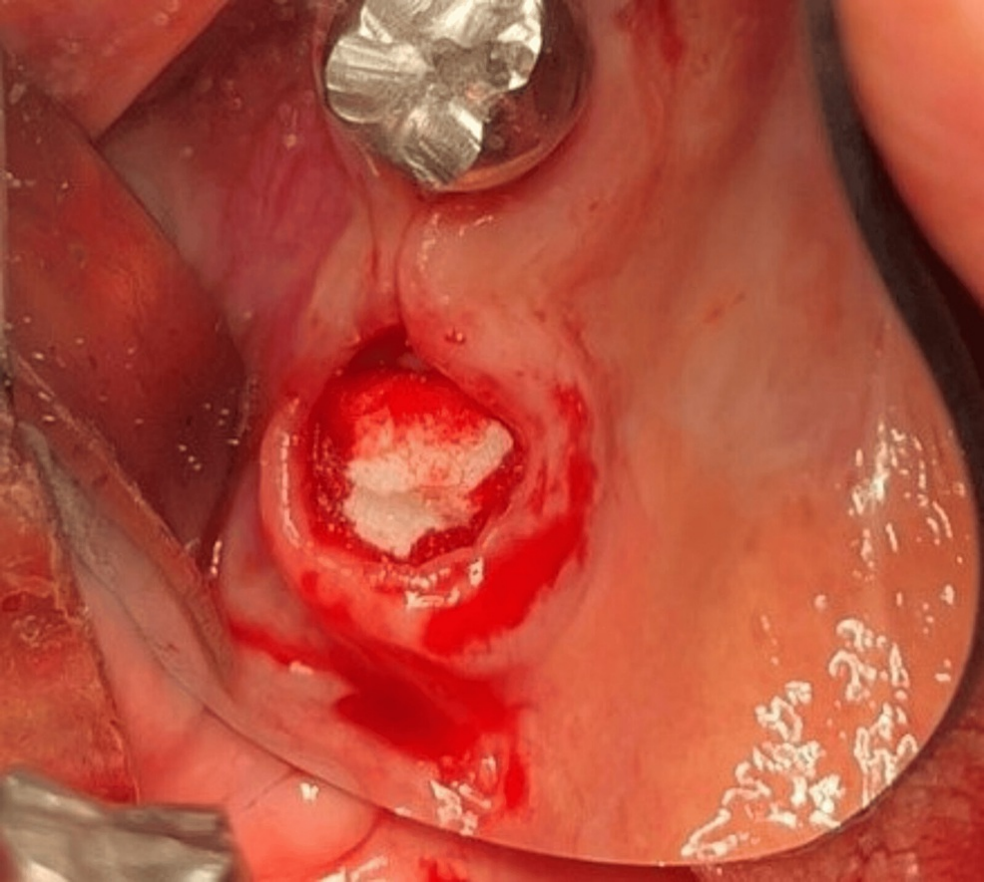
Socket filled with a collagen cone and a gelatin sponge (Case 1)

**Figure 4:**
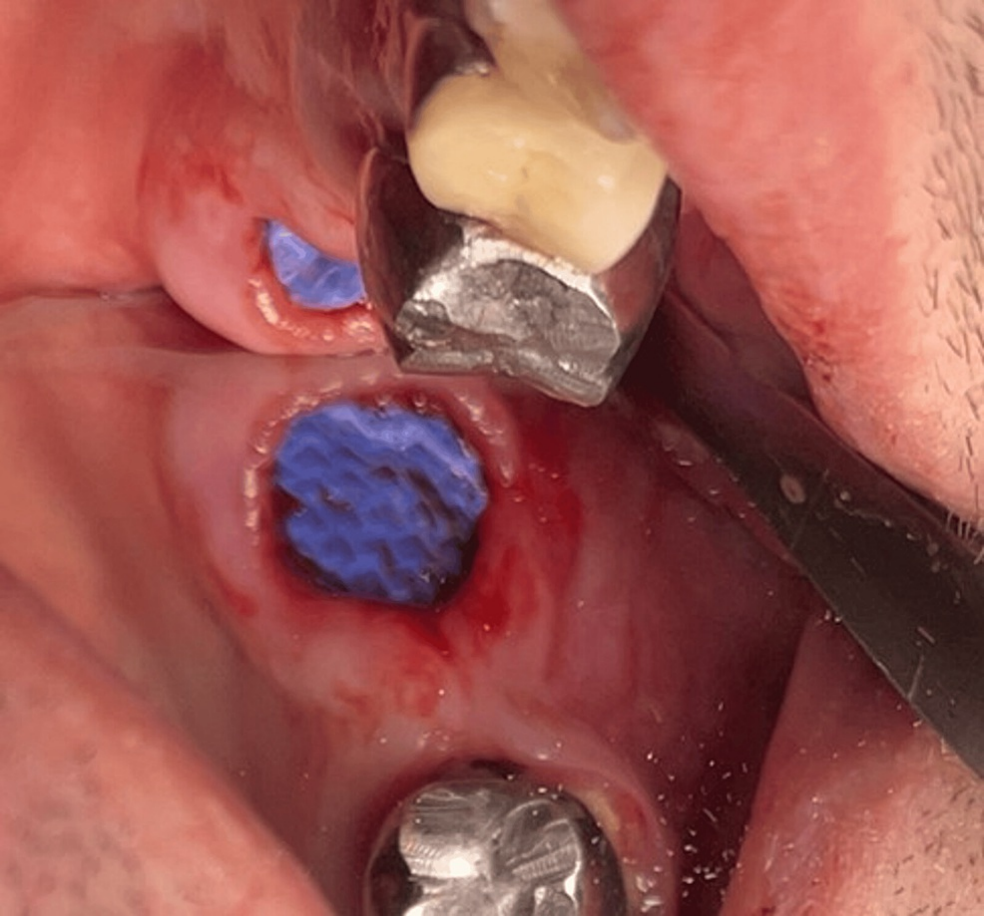
Socket sealing with a dense polytetrafluoroethylene membrane (Case 1)

**Figure 5:**
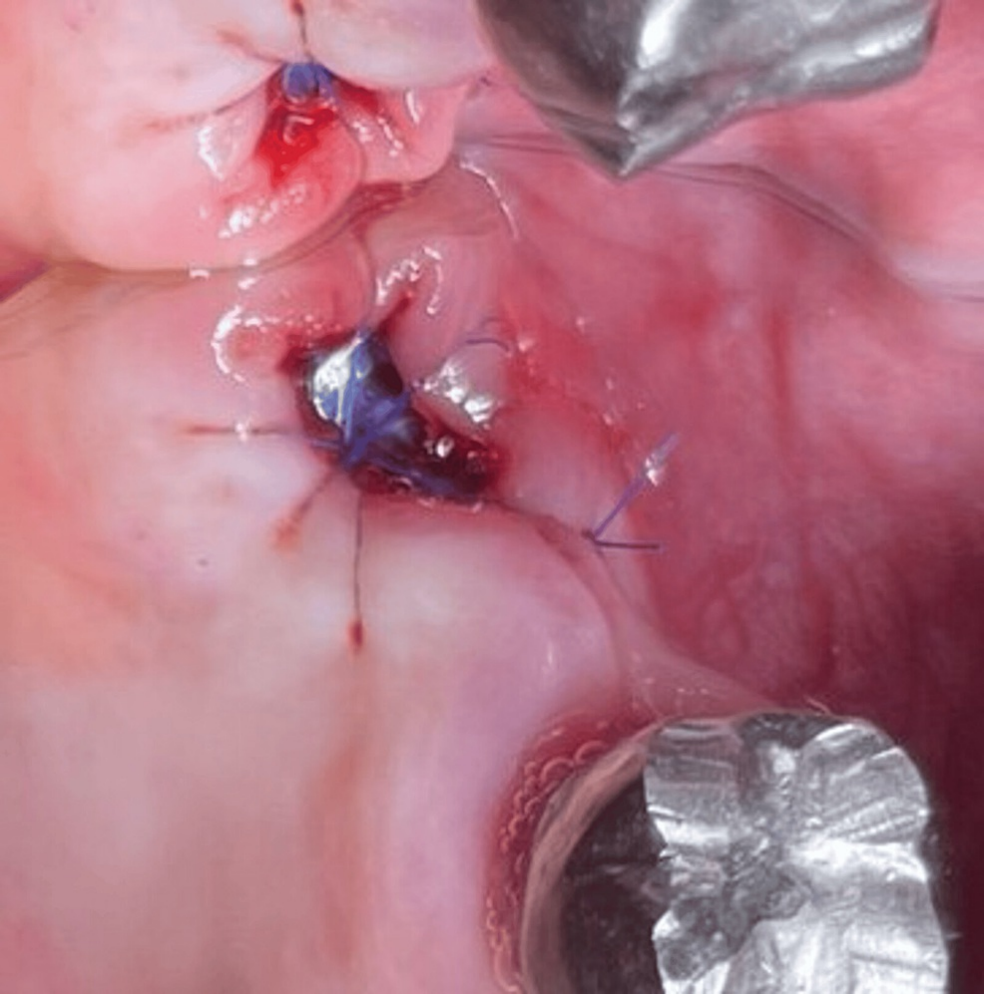
Stabilization of the dense polytetrafluoroethylene membrane with sutures (Case 1)

The patient had no current complaints concerning FDI tooth #28, although he had experienced some previous pain and discomfort a few months ago. The risk of OAC after the extraction of FDI tooth #28 and the treatment options were thoroughly explained to the patient. Two days later, an informed consent form was signed in accordance with the Helsinki Declaration, and the extractions were performed under local anesthesia with 7 ml articaine, 4%, and under monitoring by an anesthesiologist. An AOC with a diameter larger than 5 mm occurred after the extraction of FDI tooth #28 (Figure 2). The nose-blowing test was positive. The surgical protocol for the closure of the OAC consisted of curettage of the socket walls, irrigation with sterile saline solution, and placement of a collagen cone (Collacone®, Botiss Biomaterials GmbH, Zossen, Germany) and a hemostatic sponge into the socket (Figure 3). The gingival margins were undermined with a periosteal elevator to create buccal and palatal full-thickness pockets without releasing incisions. Then a non-resorbable d-PTFE (Permamem®, Botiss Biomaterials GmbH) was trimmed to cover the socket orifice, and 3-5 mm of the buccal and palatal bony walls subperiosteally, as well as to stand at 1 mm to the adjacent teeth. The membrane was inserted in the thus-prepared surgical site and stabilized by a single interrupted and an interrupted crossed mattress sutures with monofilament polyamide material (5/0) (Figure 4 and Figure 5).

The postoperative drug therapy consisted of antibacterial therapy (amoxicillin 875 mg+clavulanic acid 125 mg (twice a day for seven days) and metronidazole 500 mg (twice a day for seven days)), anti-inflammatory drugs (nimesulide 100 mg (twice a day for three days)), probiotic, antihistamine, nasal decongestant, and 0.12% chlorhexidine mouth rinse (twice daily for two weeks). The patient was given postoperative care instructions (diet and hygiene) and sinus precautions instructions.

**Figure 6:**
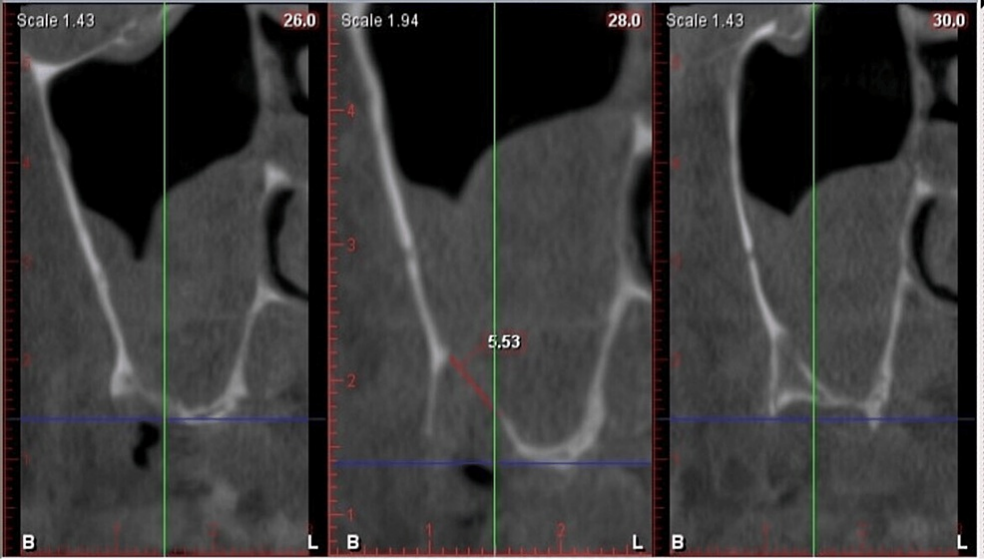
Cone-beam computed tomography after the surgery: paraxial slices (day 5) (Case 1)

**Figure 7:**
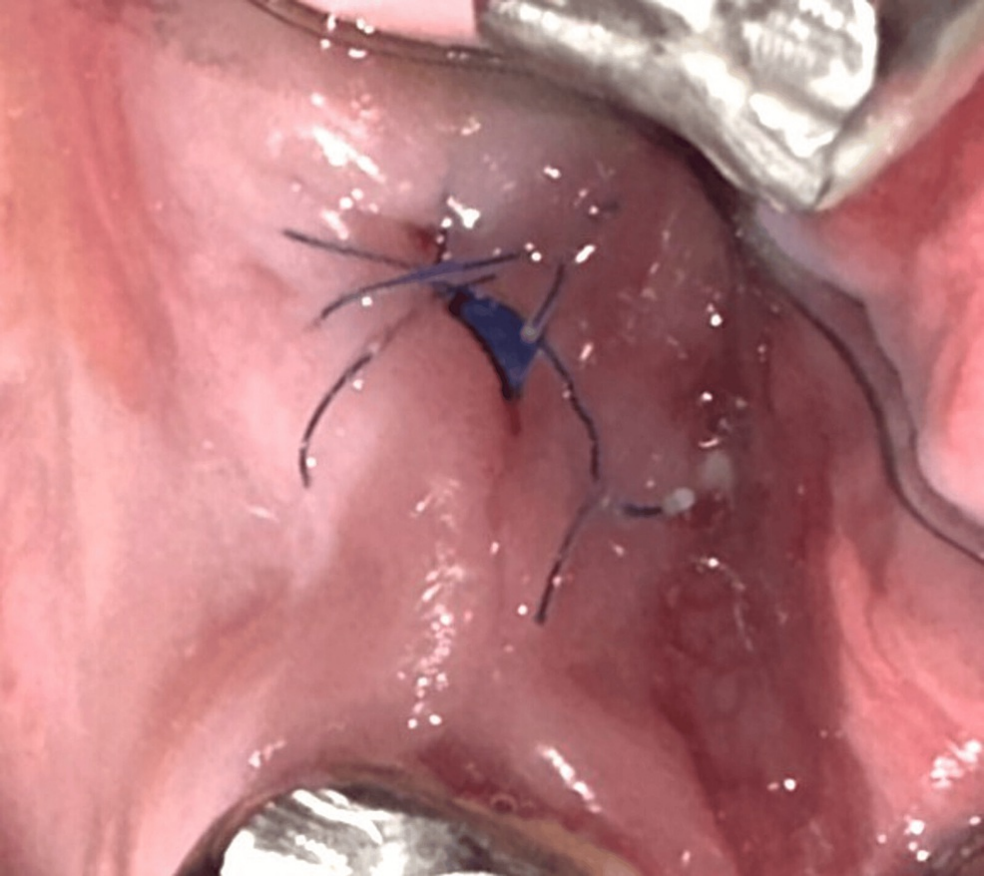
Day 5: a clinical view of the wound (Case 1)

**Figure 8:**
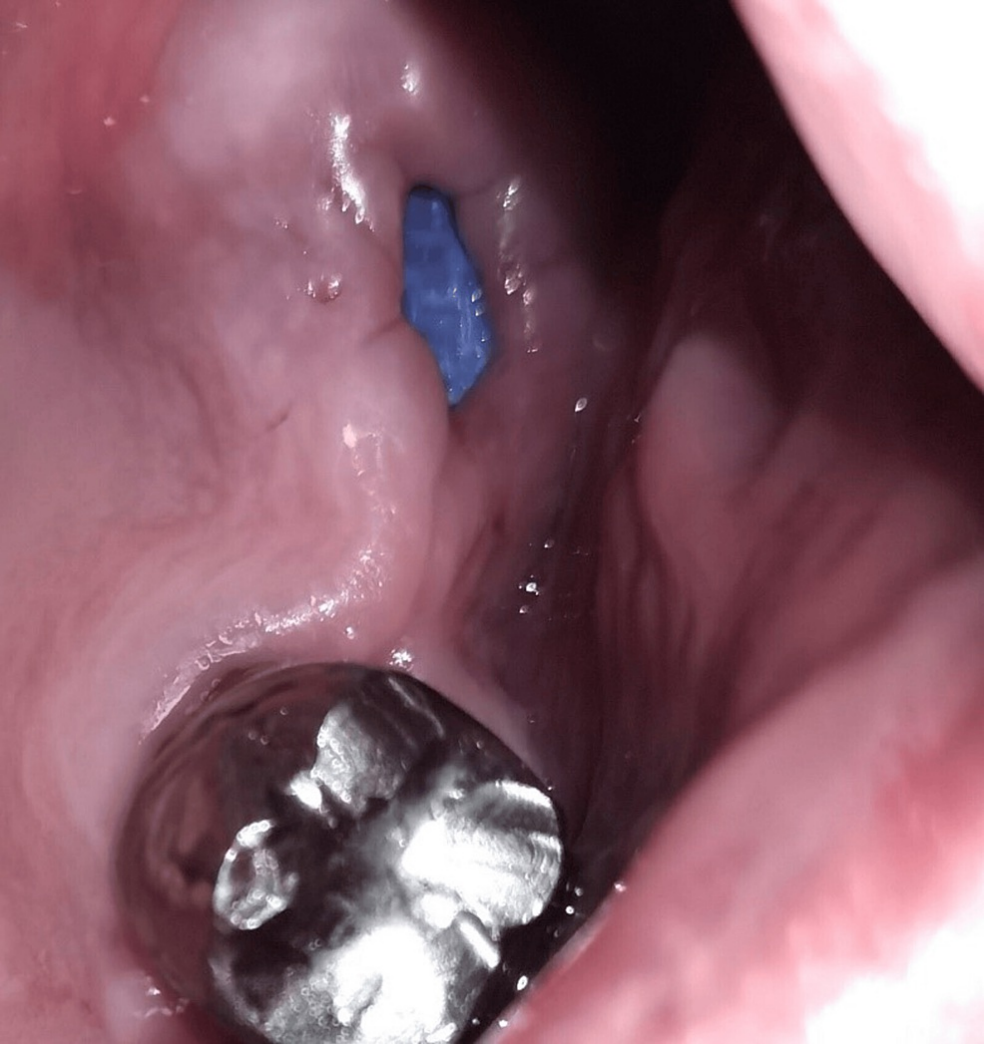
Day 14: after suture removal (Case 1)

**Figure 9:**
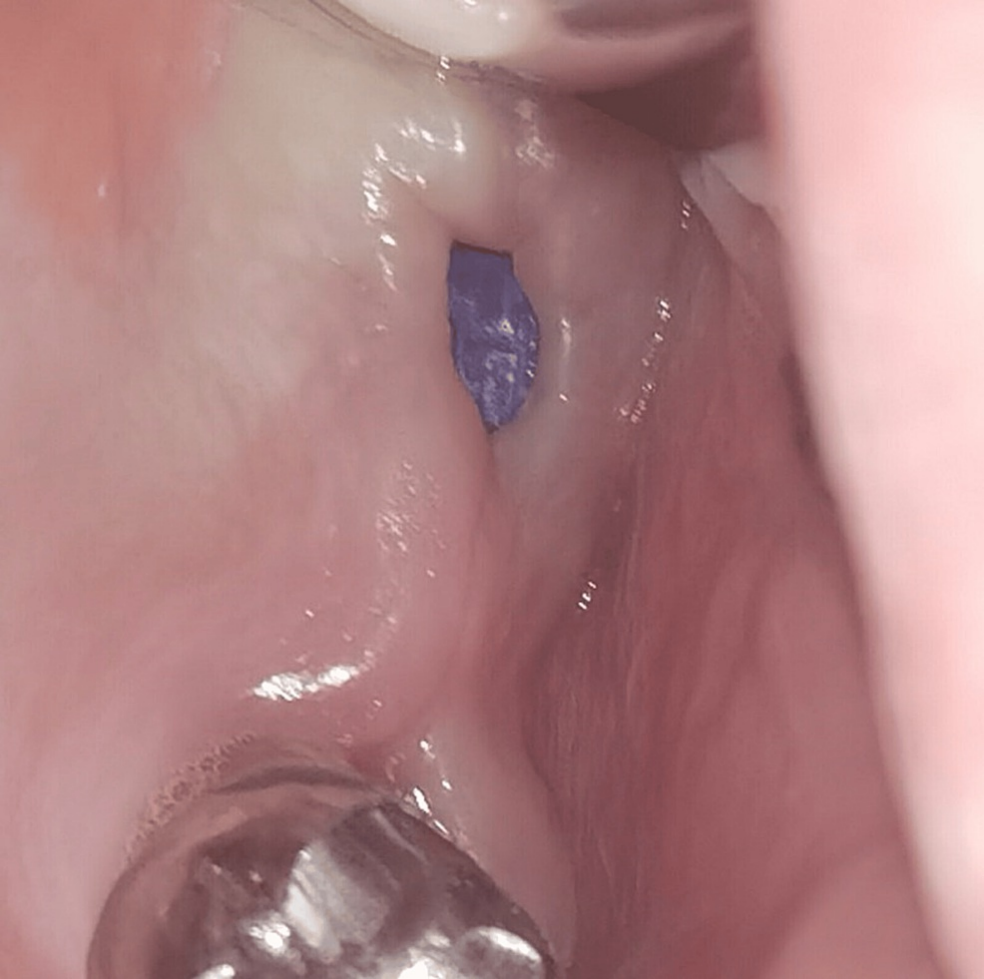
Day 28: before the removal of the membrane (Case 1)

**Figure 10:**
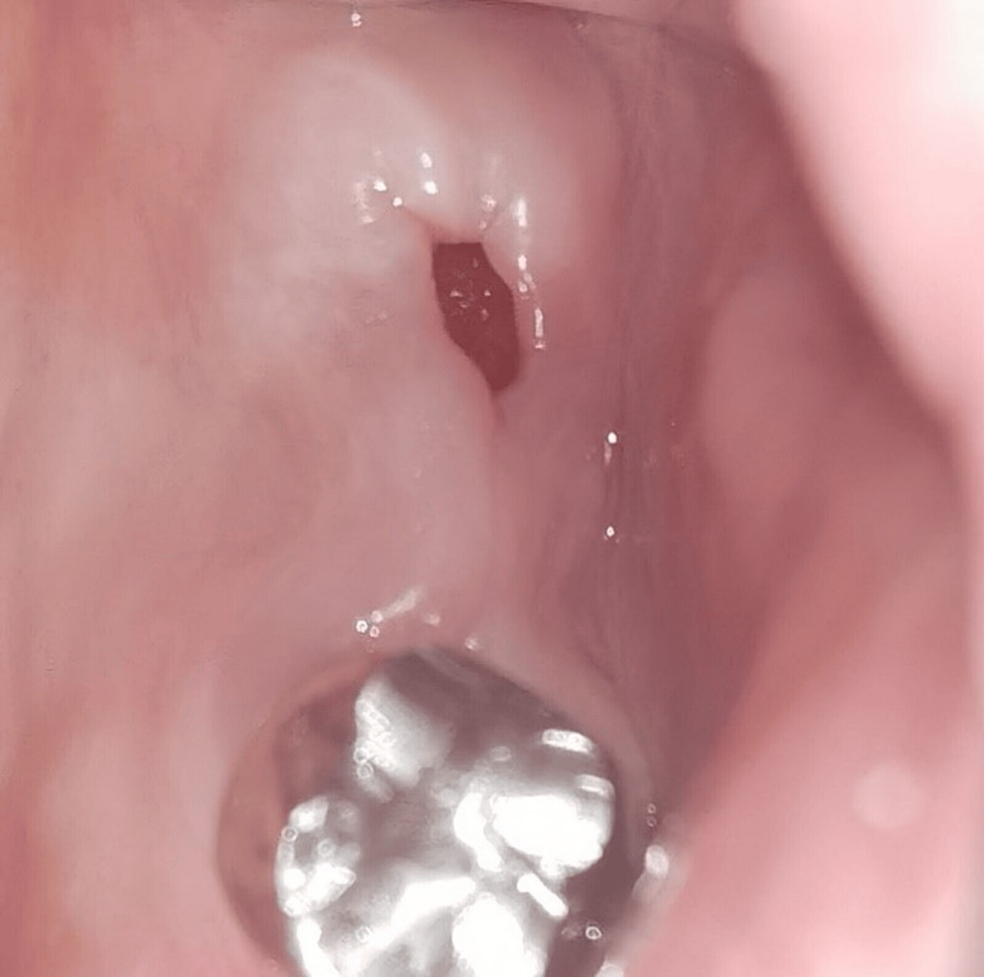
Day 28: after the removal of the membrane (Case 1)

Follow-up visits were scheduled for days 5, 14, and 28 postoperatively. On day 5, a cone-beam computed tomography (CBCT) (Figure 6) and a clinical investigation of the surgical site were performed (Figure 7). Sutures were removed on day 14 (Figure 8), and the membrane was removed on day 28 (Figure 9 and Figure 10). The healing was uneventful with a successful closure of the OAC. The patient did not come for further follow-up due to his hospitalization for the treatment of multiple myeloma.

Case 2

**Figure 11:**
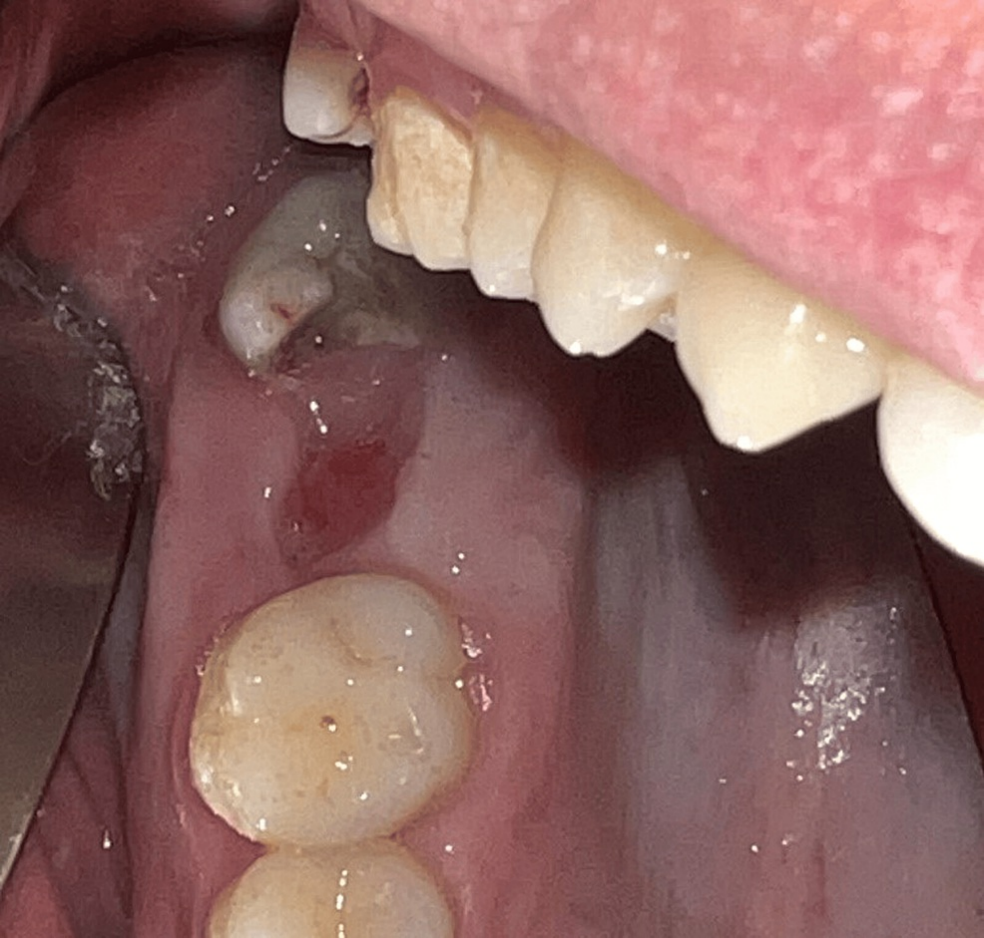
Preoperative clinical view (Case 2)

**Figure 12:**
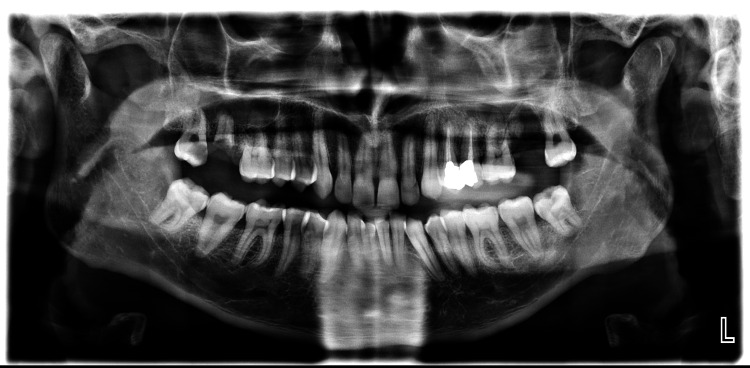
Preoperative panoramic radiograph (Case 2)

A 38-year-old man was referred to the Department of Oral Surgery in the University Medical-Dental Centre of the Medical University of Varna, Varna, Bulgaria, in March 2024 for the extraction of the upper right second molar (FDI tooth #17). He had pain in the area during mastication. The clinical examination revealed a fractured tooth below the gingival margin (Figure 11). The panoramic radiograph displayed a periapical radiolucency, which is indicative of a periapical lesion. The radiographic appearance of the lesion is consistent with a radicular cyst (Figure 12).

**Figure 13:**
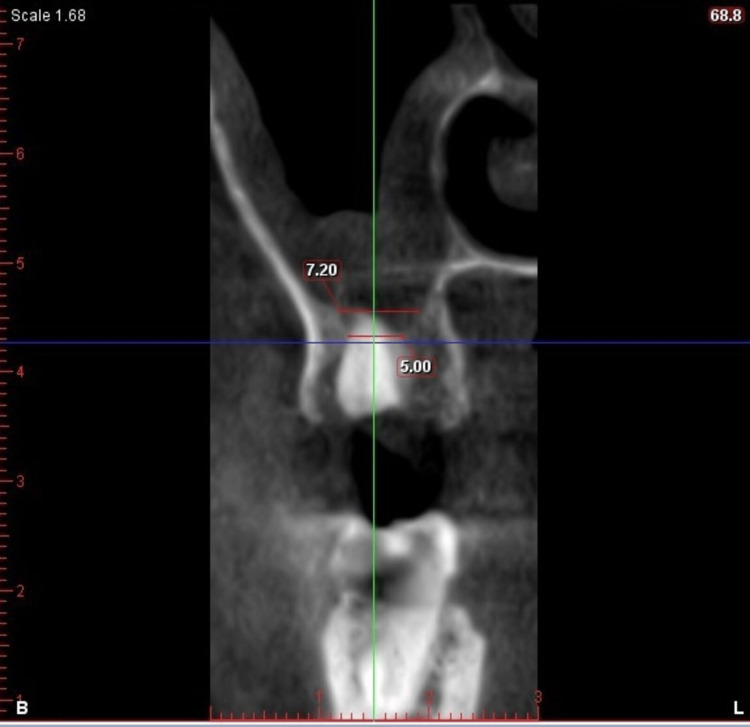
Preoperative cone-beam computed tomography image: paraxial slice (Case 2)

A CBCT scan was performed and it confirmed that the periapical pathology involved the maxillary sinus (Figure 13).

**Figure 14:**
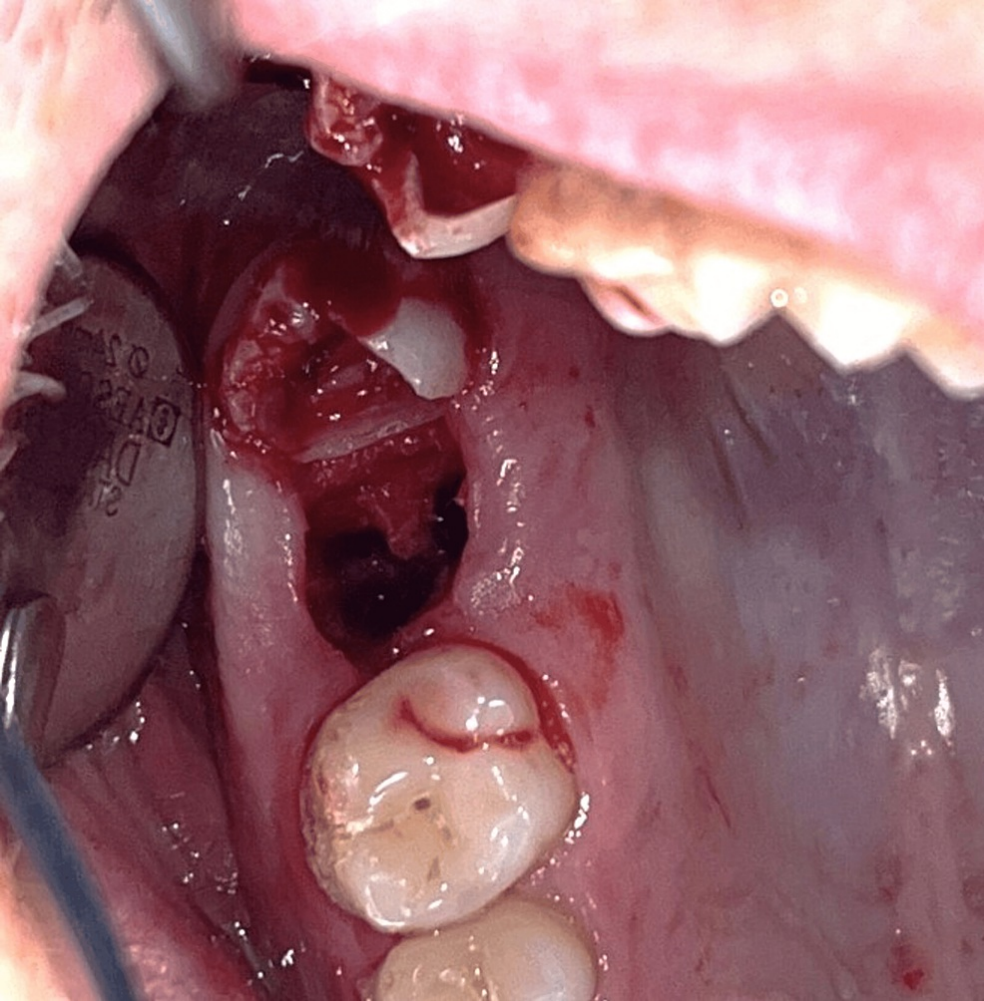
A clinical view of the oroantral communication (Case 2)

**Figure 15:**
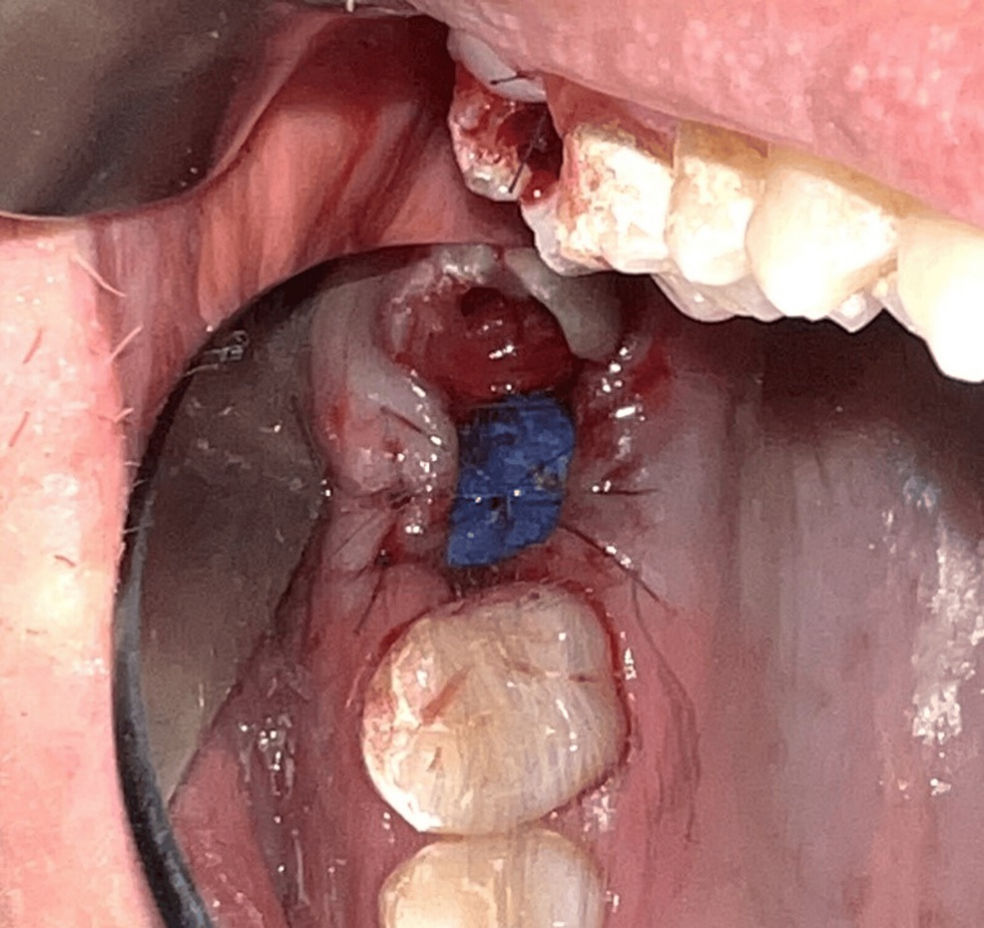
Stabilization of the dense polytetrafluoroethylene membrane with sutures (Case 2)

On the following day, an informed consent form was signed, and the tooth was extracted under local anesthesia with 1.8 ml articaine, 4%. An OAC with a diameter of 5 mm was observed (Figure 14), and the nose-blowing test was positive. Then debridement of the socket walls and irrigation with sterile saline solution were performed. The gingival margins were undermined with a periosteal elevator, and a d-PTFE membrane was placed and stabilized by a single interrupted and an interrupted crossed mattress sutures with monofilament polyamide material (5/0) (Figure 15).

The postoperative drug therapy consisted of antibacterial therapy (amoxicillin 875 mg+clavulanic acid 125 mg (twice a day for seven days) and metronidazole 500 mg (twice a day for seven days)), anti-inflammatory drugs (nimesulide 100 mg (twice a day for three days)), probiotic, antihistamine, nasal decongestant, and 0.12% chlorhexidine mouth rinse (twice daily for two weeks). The patient was given postoperative care instructions (diet and hygiene) and sinus precautions instructions.

**Figure 16:**
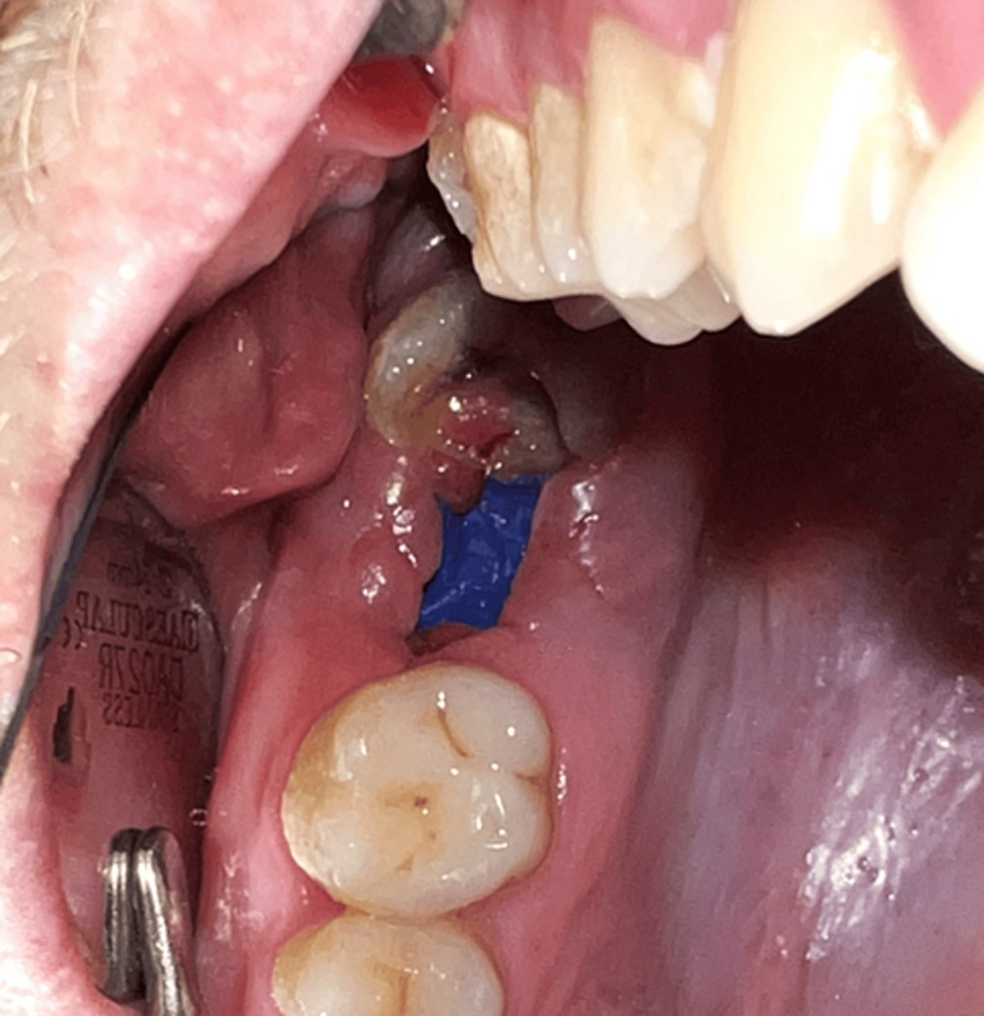
Day 14: suture removal (Case 2)

**Figure 17:**
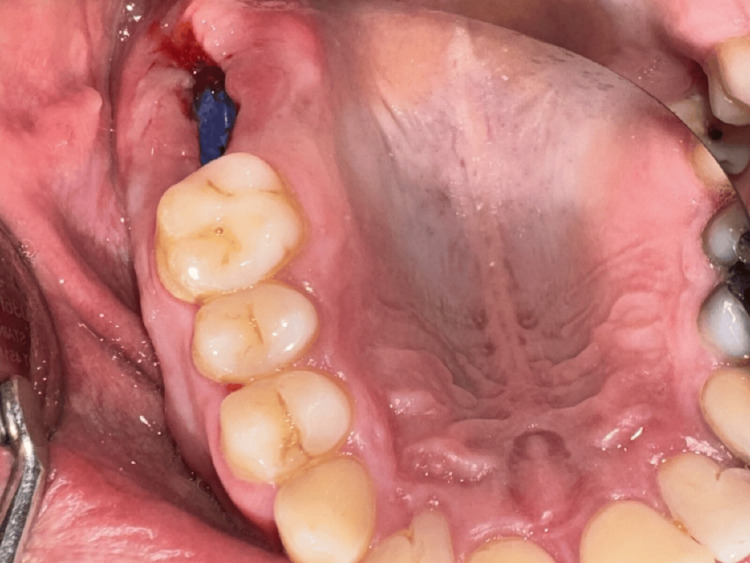
Day 28: before the removal of the membrane (Case 2)

**Figure 18:**
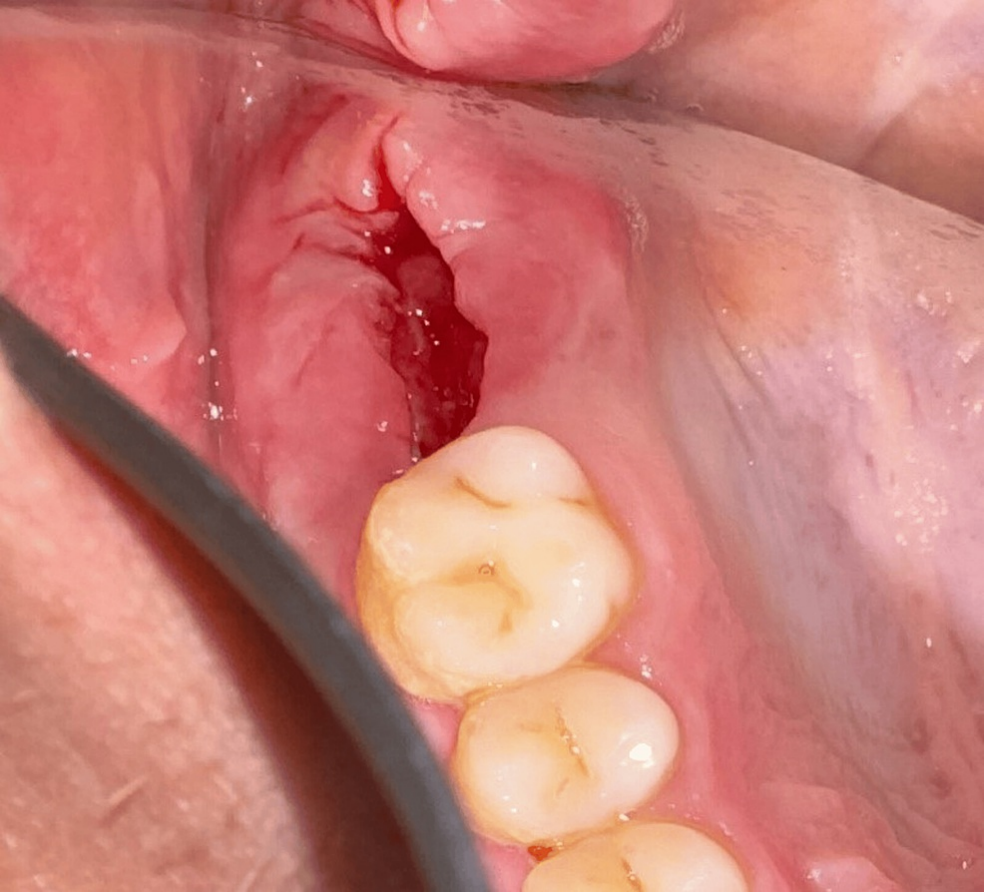
Day 28: after the removal of the membrane (Case 2)

**Figure 19:**
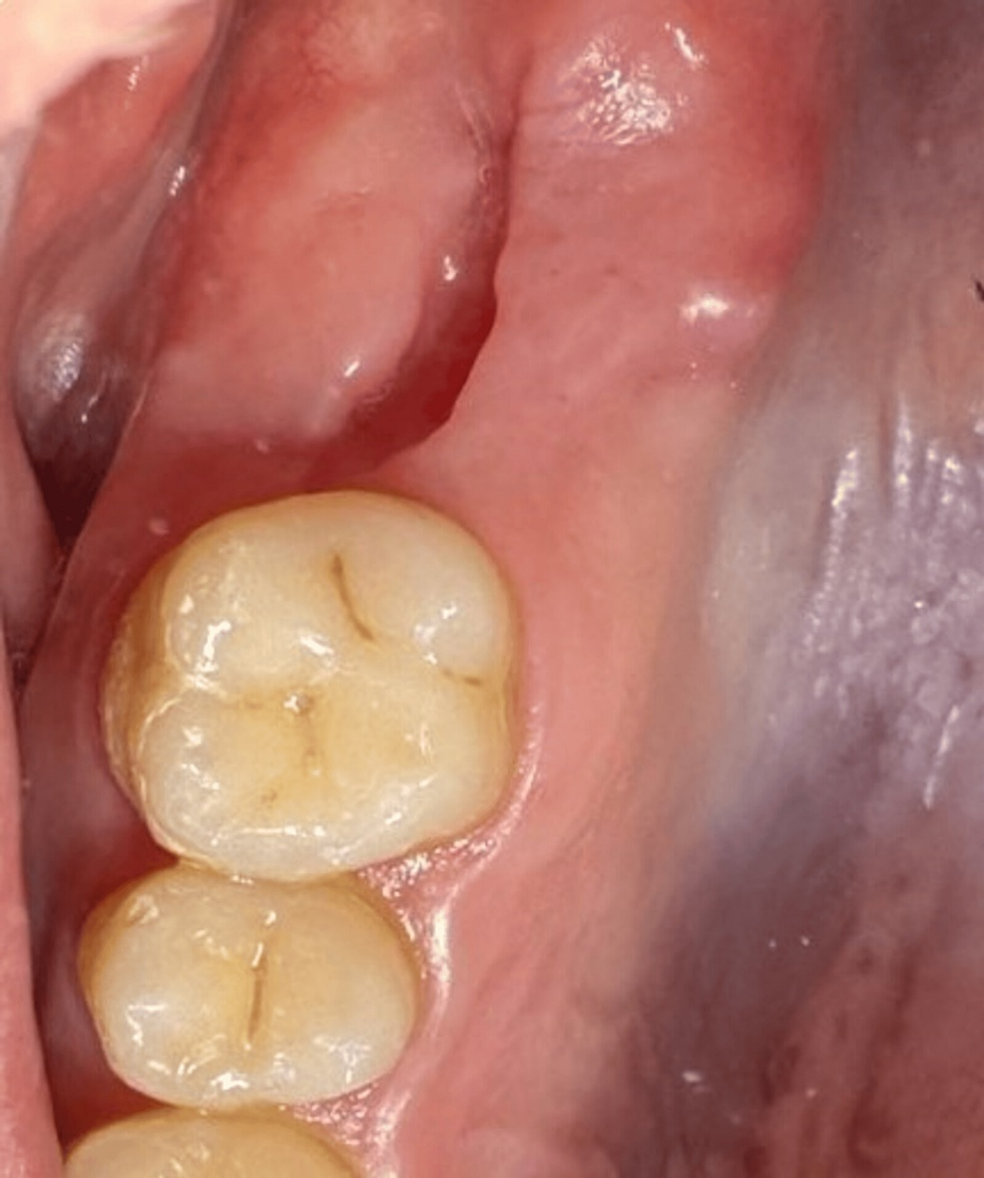
Day 35: postoperative follow-up (Case 2)

Follow-up visits were scheduled for days 14 (sutures removal) (Figure 16), 28 (membrane removal) (Figure 17 and Figure 18), and 35 (Figure 19). Healing was uneventful with a successful closure of the OAC.

## Discussion

OACs are relatively common complications after extractions of maxillary molars, premolars, and sometimes canines [5]. If left untreated, they can lead to OAFs. The latter act as pathological pathways for bacteria that can spread into the maxillary sinus and cause maxillary sinusitis or even pansinusitis in up to 60% of the cases [1]. Therefore, an immediate and effective closure of the OAC is necessary. If blood clot retention can be easily achieved, OAC can heal spontaneously. However, other situations, such as OAC larger than 3 mm, shallow sockets, missing bony walls, etc., require surgical treatment of the defect [6].

Among the most common techniques are the methods that utilize buccal advancement flaps, palatal advancement flaps, rotational advancement flaps, closure with the buccal fat pad, and so on [7]. However, they have a lot of disadvantages, such as shallowing of the buccal vestibule, loss of attached gingiva, displacement of the mucogingival junction, and difficult implant and prosthodontic rehabilitation [8]. In addition, these methods are more technically challenging and are often associated with postoperative discomfort, pain, facial edema, ecchymosis, hematoma, and so on. Moreover, if these methods fail, the options for further treatment are reduced.

Recently, techniques that utilize bone substitutes and tissue regeneration materials have gained popularity. Such a method is the open barrier technique with d-PTFE membranes. These membranes are made of 100% synthetic material which is non-resorbable, biocompatible, and biologically inert [9,10]. Their high density and microporosity (with a pore diameter of less than 0.3 µm) make them an excellent barrier for epithelial cells and bacteria [10,11]. Their major advantage is that primary closure is not necessary and they can be left exposed. These properties allow for socket coverage without alterations in the soft tissue profile. The membranes have been successfully utilized for alveolar ridge preservation [9,12]. Their use for the treatment of OAC decreases the need for soft tissue flap mobilization and the subsequent disadvantages.

Barrier membranes have been widely used for guided tissue regeneration in oral surgery and implant dentistry [12-14]. Lee was the first to describe the closure of OAC with a d-PTFE membrane [10]. He mobilized soft tissue flaps, covered the socket orifice with the membrane, and repositioned the flaps in a tension-free manner.

Scavia et al. have recently published a case series of ridge preservation combined with the closure of OACs using an open barrier technique. The OACs had diameters of 2-5 mm. The communications were first covered with collagen fleece, and then the alveolar ridges were reconstructed with a bone substitute and covered with d-PTFE membranes [15].

The clinical results of our study confirm that the open barrier technique can be successfully used for the closure of OACs. In one of the cases, the communication was larger than 5 mm, yet the healing was uneventful. However, further investigation is necessary to evaluate the method, the alterations of the alveolar ridge, and the surrounding soft tissues, as well as to find which materials provide optimal ridge preservation.

## Conclusions

Among the most commonly used, safe, and time-proven methods for the closure of OACs are those with advancement flaps. Although most of these techniques have excellent success rates, their major drawback is the change of the prosthetic field: reduction of the amount of attached gingiva, the depth of the vestibule, the displacement of the mucogingival border, cicatricial shrinkage, etc. Therefore, improving the well-known methods or introducing new methods is necessary to achieve optimal results, reduce the risk of failure, and eliminate the disadvantages mentioned above.

## References

[1] Mainassara Chekaraou S, Benjelloun L, El Harti K (2021). Management of oro-antral fistula: two case reports and review. Ann Med Surg (Lond).

[2] Dym H, Wolf JC (2012). Oroantral communication. Oral Maxillofac Surg Clin North Am.

[3] Bhalla N, Sun F, Dym H (2021). Management of oroantral communications. Oral Maxillofac Surg Clin North Am.

[4] Oliva S, Lorusso F, Scarano A, D'Amario M, Murmura G (2024). The treatment and management of oroantral communications and fistulas: a systematic review and network metanalysis. Dent J (Basel).

[5] Georgiev D, Peev S, Arnautska H (2015). Relationship between root apices of maxillary posterior teeth and the maxillary sinus floor in patients from the Varna region. J Med Dent Pract.

[6] Hernando J, Gallego L, Junquera L, Villarreal P (2010). Oroantral communications. A retrospective analysis. Med Oral Patol Oral Cir Bucal.

[7] Bereczki-Temistocle DL, Gurzu S, Jung I (2022). Selecting the best surgical treatment methods in oro-antral communications. Int J Environ Res Public Health.

[8] Parvini P, Obreja K, Begic A, Schwarz F, Becker J, Sader R, Salti L (2019). Decision-making in closure of oroantral communication and fistula. Int J Implant Dent.

[9] Yotsova R, Peev S, Georgiev Georgiev, T T (2021). Alveolar ridge preservation using dense polytetrafluoroethylene membranes. A review article. Scr Sci Med Dent.

[10] Lee C (2016). Use of a non-resorbable DPTFE membrane to close an oroantral communication of the posterior maxilla after tooth extraction: a case report. Ann Otolaryngol Rhinol.

[11] Yankov YG (2023). Socket preservation and guided bone regeneration: prerequisites for successful implant dentistry. Cureus.

[12] Aprile P, Letourneur D, Simon-Yarza T (2020). Membranes for guided bone regeneration: a road from bench to bedside. Adv Healthc Mater.

[13] Peev S, Gusiyska A, Sabeva E (2016). Guided bone regeneration and simultaneous implant placement. Int J Sci Res.

[14] Yang Z, Wu C, Shi H, Luo X, Sun H, Wang Q, Zhang D (2022). Advances in barrier membranes for guided bone regeneration techniques. Front Bioeng Biotechnol.

[15] Scavia S, Audino E, Salgarello S (2024). Ridge preservation combined with open barrier membrane technique in case of post-extractive oro-antral communication: a case series retrospective study. J Oral Implantol.

